# Zhilong Huoxue Tongyu capsule attenuates intracerebral hemorrhage induced redox imbalance by modulation of Nrf2 signaling pathway

**DOI:** 10.3389/fphar.2023.1197433

**Published:** 2023-06-07

**Authors:** Maryam Mazhar, Guoqiang Yang, Houping Xu, Yulin Liu, Pan Liang, Luyin Yang, Roman Spáčil, Hongping Shen, Dechou Zhang, Wei Ren, Sijin Yang

**Affiliations:** ^1^ National Traditional Chinese Medicine Clinical Research Base and Drug Research Center, the Affiliated Traditional Chinese Medicine Hospital of Southwest Medical University, Luzhou, China; ^2^ Institute of Integrated Chinese and Western Medicine of Southwest Medical University, Luzhou, China; ^3^ Research Center for Integrated Chinese and Western Medicine, the Affiliated Traditional Chinese Medicine Hospital of Southwest Medical University, Luzhou, Sichuan, China; ^4^ Molecular Imaging and Therapy Research Unit, Center of Radiation Research and Medical Imaging, Department of Radiologic Technology, Faculty of Associated Medical Sciences, Chiang Mai University, Chiang Mai, Thailand; ^5^ Preventive Treatment Center, the Affiliated Traditional Chinese Medicine Hospital of Southwest Medical University, Luzhou, China; ^6^ Chengdu University of Traditional Chinese Medicine, Chengdu, Sichuan, China; ^7^ The Czech Center for Traditional Chinese Medicine, Olomouc, Czechia

**Keywords:** intracerebal haemorrhage, traditional Chinese medicine, Nrf2 signalling, redox imbalance, antioxidants

## Abstract

**Background:** One of the severely debilitating and fatal subtypes of hemorrhagic stroke is intracerebral hemorrhage (ICH), which lacks an adequate cure at present. The *Zhilong Huoxue Tongyu* (ZLHXTY) capsule has been utilized effectively since last decade to treat ICH, in some provinces of China but the scientific basis for its mechanism is lacking. *Purpose:* To investigate the neuroprotective role of ZLHXTY capsules for ICH-induced oxidative injury through the regulation of redox imbalance with the Nrf2 signaling pathway.

**Methods:** Autologous blood injection model of ICH in C57BL/6J mice was employed. Three treatment groups received ZLHXTY once daily through oral gavage at doses 0.35 g/kg, 0.7 g/kg, and 1.4 g/kg, started after 2 h and continued for 72 h of ICH induction. The neurological outcome was measured using a balance beam test. Serum was tested for inflammatory markers IL-1β, IL-6, and TNF-α through ELISA, oxidative stress through hydrogen peroxide content assay, and antioxidant status by total antioxidant capacity (T-AOC) assay. Nuclear extract from brain tissue was assayed for Nrf2 transcriptional factor activity. RT-qPCR was performed for Nfe2l2, Sod1, Hmox1, Nqo1, and Mgst1; and Western blotting for determination of protein expression of Nrf2, p62, Pp62, Keap, HO1, and NQO1. Fluoro-jade C staining was also used to examine neuronal damage.

**Results:** ZLHXTY capsule treatment following ICH demonstrated a protective effect against oxidative brain injury. Neurological scoring showed improvement in behavioral outcomes. ELISA-based identification demonstrated a significant decline in the expression of serum inflammatory markers. Hydrogen peroxide content in serum was found to be reduced. The total antioxidant capacity was also reduced in serum, but the ZLHXTY extract showed a concentration-dependent increase in T-AOC speculating at its intrinsic antioxidant potential. Nrf2 transcriptional factor activity, mRNA and protein expression analyses revealed normalization of Nrf2 and its downstream targets, which were previously elevated as a result of oxidative stress induced by ICH. Neuronal damage was also reduced markedly after ZLHXTY treatment as revealed by Fluoro-jade C staining. *Conclusion:* ZLHXTY capsules possess an intrinsic antioxidant potential that can modulate the ICH-induced redox imbalance in the brain as revealed by the normalization of Nrf2 and its downstream antioxidant targets.

## Highlights

• Nrf2 signaling activates in response to oxidative stress after ICH-induced brain injury.
• ZLHXTY capsules possess an intrinsic antioxidant potential that regulates the ICH-induced redox imbalance in the brain.
• ZLHXTY capsules mediate neuroprotection through anti-inflammatory and anti-oxidant pathways.

## Introduction

Among the subtypes of hemorrhagic stroke, intracerebral hemorrhage (ICH) is the most seriously debilitating and fatal, associated with high morbidity and mortality, inflicting  5 million people each year worldwide (An et al., 2017). Primarily, ICH injury is due to the rupture of blood vessels releasing massive blood volumes into the surrounding parenchyma, causing high intracranial pressure and inflammatory injury to the brain tissue (Keep et al., 2012). Preclinical and clinical studies have evidenced the key role of reactive oxygen species (ROS) mediated oxidative stress and inflammation in the progression of ICH-induced early brain injury (Lan et al., 2019). However, inflammatory responses can be modulated by oxidative stress through the activation of nuclear factor erythroid 2-related factor 2 (Nrf2) (He et al., 2020). Nrf2 is known to be a master regulator of the antioxidant system in our body and a fundamental transcriptional factor for genes regulated by antioxidant response element (ARE). This system is triggered on exposure to oxidative stress response to protect many organs including the brain, by the upregulation of cytoprotective and antioxidant genes and regulation of the cell survival (Ma, 2013; Saha and Buttari, 2020). Several studies have reported the activation of Nrf2 signaling after induction of intracerebral hemorrhage to provide protection against aggravation of early brain injury (Zhao et al., 2007; Zhao and Aronowski, 2013; Zhao et al., 2015; Chen-Roetling and Regan, 2017).

The lack of effective therapeutic options for ICH in clinics is impelling researchers to develop potent remedies (Liddle et al., 2020). Recently, the use of traditional Chinese medicine (TCM) has become popular as a sole or adjunct therapy for the treatment of various illnesses not only in China but all around the globe (Zhao et al., 2021). In 2019, TCM was accepted in the 11th revision of the International Statistical Classification of Diseases and Related Health Problems (ICD-11) as an effective therapeutic category (Lam et al., 2019). Moreover, in order to accomplish universal health coverage (UHC) as a Sustainable Development Goal 3 (SDG 3), the allocation of traditional and complementary medicines in mainstream healthcare and medical services has also been encouraged by World Health Organization (World Health Organization, 2019). Zhilong Huoxue Tongyu (ZLHXTY) capsule is a TCM formula containing a mixture of five Chinese medicines: *Pheretima Aspergillum* (E. Perrier), *Hirudo nipponica* Whitman, *Astragalus membranaceus* Fisch. ex Bunge, *Sargentodoxa cuneata* (Oliv.) Rehder and E.H. Wilson, and *Cinnamomum cassia* (L.) J. Presl (Table 1). Interestingly, antioxidant activity is common among all of these components (Yang et al., 2012; Rao and Gan, 2014; Durazzo et al., 2021; Li et al., 2021; Samuel et al., 2021; Zhang et al., 2021; Xu et al., 2022). According to TCM theory, the combination of these five drugs work in concert to reduce inflammation, resist oxidation, and fight against aging by enriching Qi, eliminating phlegm and blood stasis, and promoting blood circulation; thereby suitable for the treatment of stroke, atherosclerosis, and other related cardiovascular and cerebrovascular conditions (Liang et al., 2021).

**TABLE 1:** Composition of Zhilong Huoxue Tongyu capsule.

Components	1	2	3	4	5
Scientific Name	*Hirudo nipponica* Whitman	*Pheretima aspergillum* (E. Perrier)	*Astragalus membranaceus* Fisch. ex Bunge	*Cinnamomum cassia* L.) J. Presl	*Sargentodoxa cuneata* (Oliv.) Rehder and E.H. Wilson
Synonyms	Not applicable	*Amynthas aspergillum* (Perrier)	*Astragalus mongholicus* Bunge	*Neolitsea cassia* L.) Kosterm.	*Holboellia cuneata* Oliv.
English Name	Leech	Earthworm	Astragalus	Cassia	Sargentgloryvine
Chinese Name	ShuiZhi	Guang Dilong	Huang Qi	GuiZhi	Da XueTeng
Family	Hirudideae	Megascolecidae	Fabaceae	Lauraceae	Lardizabalaceae
Parts Used	Dried whole animal	Dried whole animal	Dried Roots	Dried Stem/Twig	Dried Stem/Twig
Dry Weight in ZLHXTY Capsule (grams)	0.32	1.7	2.3	0.86	1.7
Source	Chengdu Renjihong Pharmaceutical Co., Ltd.	Chengdu Renjihong Pharmaceutical Co., Ltd.	Sichuan Jinfang Biomedical Technology Co., Ltd.	Sichuan Tianzhi Traditional Chinese Medicine Co., Ltd.	Sichuan Jinfang Biomedical Technology Co., Ltd.
Batch Number	190101	190301	190501	20030101	190402
Origin	Anhui, China	Guangxi, China	Gansu, China	Anhui, China	Guangxi, China

Our previous research has demonstrated that ZLHXTY capsules inhibit inflammation after ICH by regulating the NFкB pathway (Mazhar et al., 2022). In this study, we further investigate the extensive mechanisms of the ZLHXTY capsule for ICH therapy, by exploring its modulatory role on the redox environment of the Nrf2 signaling pathway, during the early stages of ICH.

## Materials and methods

### Materials

Zhilong Huoxue Tongyu (ZLHXTY) capsules were obtained from the pharmacy department of the Affiliated TCM Hospital of Southwest Medical University, Luzhou, Sichuan, China. ZLHXTY capsule is a hospital preparation of the Affiliated Traditional Chinese Medicine Hospital, Southwest Medical University, Luzhou China. The formula is adapted by Prof. Sijin Yang according to the Buyang Huanwu decoction method, which is approved by the State Intellectual Property Office of the People’s Republic of China (Patent No. 200810147774.1). Briefly, the Chinese herbs were obtained from various sources (Table 1) that were authenticated by Prof. Qingrong Pu, Director of TCM Preparation Room of The Affiliated Chinese Medicine Hospital of Southwest Medical University. Dried whole bodies of *H. nipponica* Whitman (0.32g) and *Pheretima aspergillum* (E. Perrier) (1.7g) were soaked in 2.85 ml of 60% ethanol for 7 days, followed by filtration and collection of filtrate A. The three herbal components, i.e., dried roots of *A. membranaceus* Fisch. ex Bunge (2.3g), dried twigs of *C. cassia* L.) J. Presl (0.86g) and *S. cuneata* (Oliv.) Rehder and E.H. Wilson (1.7g), were decocted twice with 13.51 ml of water for 1 h each time and the volatile components were also collected. The decoctions collected in two steps were combined together and filtered. The filtrate was concentrated at 80 °C to a relative density of 1.05 ≤ 1.10. Later, ethanol was added to the concentrate to make the alcohol content up to 50%, and was kept standing for 12 h followed by filtration. The resulting filtrate B was combined with the ethanolic infusion A. After the alcohol was recovered, the resulting mixture was boiled and concentrated at 80 °C to a relative density of 1.20. The concentrate was added with 0.2g of dextrin as an excipient, boiled, granulated, and crushed into a fine powder and sprayed and mixed with volatile components collected previously. Finally, 0.4g of the powdered mixture was filled in a capsule shell to make one capsule dosage form. The whole drug extract ratio (DER) is 12.9%. All the procedures were performed following good laboratory practices (GLP). The HPLC-HR-MS analysis of the ZLHXTY capsule has been performed previously and can be referred to in our previous publication (Mazhar et al., 2022) (Supplementary Table S1; Supplementary Figure S1).

### Animals

Male C57BL/6J mice weighing 20–22g, and 7–8 weeks old, were raised in the controlled environment of an animal house facility regulated at a temperature of 22 °C, and humidity of 55%, with alternate 12-h light and dark cycles. All animals were provided with normal rodent food and plain water *ad libitum*. The study design was approved by the Animal Research Committee of Southwest Medical University, Luzhou, China; and all the procedures were conducted in accordance with the National Institute of Health (NIH) Guide for the Care and Use of Laboratory Animals.

### Intracerebral hemorrhage model

The autologous blood injection model of ICH was employed in this study. Briefly, animals were anesthetized with sodium pentobarbital (dose 50 mg/kg) intraperitoneal injection. The mouse head was fixed in a stereotaxic frame with a small incision made on the skin. A sterile cotton bud soaked in 30% H_2_O_2_ was applied on the surface of the skull to remove the periosteum. At co-ordinates of 2.5 mm lateral and 0.2 mm anterior to the bregma, a 1-mm burr hole was drilled in the right striatum, through which a volume of 25 μL autologous blood obtained from the tail vein was injected at a rate of 5 μL/min, with a needle insertion depth of 3 mm. To prevent backflow of blood, the needle was held steady for 5 min after the completion of injection and then withdrawn gently. The incision was sutured in aseptic conditions. The animals were observed for vital signs and maintained in a warm environment at 37 °C until consciousness was regained.

### Treatment groups

There were five experimental groups with six mice in each, distributed randomly. The experiments were repeated three independent times. The groups were as follows i) normal control, ii) ICH model group, iii) Low dose (0.35 g/kg) ZLHXTY-LD, iv) Medium dose (0.7 g/kg) ZLHXTY-MD, and v) High dose (1.4 g/kg) ZLHXTY-HD.

The first ZLHXTY dose was given by oral gavage after 2 h of ICH induction, followed by once daily dosing for three consecutive days. The mice in the normal and ICH model groups were orally gavaged with normal saline. The animals from respective groups were sacrificed after 24 and 72 h, for subsequent assays and molecular analysis. From the previous experiments, the dose-dependent effect of ZLHXTY capsules on ICH recovery has been established. To be concise, only the highest dose data with the best effective results are presented here, except for neurological scoring outcomes showing the three dosing groups of ZLHXTY capsule treatment.

### Balance beam test with neurological scoring

The animals were examined for neurological scoring on a balance beam (Feeney et al., 1982; Liu et al., 2020) 2 days prior to the induction of ICH and continued after 3 h s, 24 h s, 48 h s, and 72 h s of ICH induction. Briefly, the mice were allowed to cross the 2 cm long beam, and the latency period from the starting point to the terminal box was noted, within 60 s of the maximum limit (Liu et al., 2020). The general behavior and gait of the animal while walking on the beam such as the number of paw slips were also recorded according to the scoring criteria (Table 2) (Feeney et al., 1982). The average score was calculated from the observations of two independent observers.

**TABLE 2:** Neurological scoring scale for beam walking test^a^.

Score	Performance on the beam
7	Traverses beam normally with both affected paws on horizontal beam surface, neither paw ever grasps the side surface, and there are no more than two footslips; toe placement style is the same as preinjury
6	Traverses beam successfully and uses affected limbs to aid >50% of steps along beam.
5	Traverses beam successfully but uses affected limbs in <50% of steps along beam.
4	Traverses beam and, at least once, places affected limbs on horizontal beam surface.
3	Traverses beam by dragging affected hindlimbs.
2	Unable to traverse beam but places affected limbs on horizontal beam surface and maintains balance for ≥5 s.
1	Unable to traverse beam; cannot place affected limbs on horizontal beam surface.

^a^ Adapted from the method of Feeney et al. (25) used to evaluate unilateral lesions of sensory cortex in rats.

### Animal surgery and specimen preparation

Following the ZLHXTY treatment regimen for 1 and 3 days, the mice in respective groups were euthanized with sodium pentobarbital overdose for dissection. A cardiac puncture was performed to collect blood in a vacutainer that was centrifuged to obtain serum. The brain tissues were collected in 1.5 ml eppendorf tubes and were snap-frozen by immersion in liquid nitrogen. Both the serum and brain tissue samples were stored at −80 °C for later use.

### ELISA for IL-1β, IL-6 and TNF-α

Sera were assayed for IL-1β, IL6, and TNF-α following the manufacturer’s procedure provided by commercially available ELISA kits (Cat# PI301, PI326, PT512) purchased from Beyotime Biotechnology, Shanghai, China. Briefly, 100 µl of each six standard dilutions, standard diluent buffer, and samples were added respectively into the wells of the ELISA plate and incubated for 2 h at room temperature. Then, the wells were rinsed 5x with washing solution, followed by incubation in 100 µl biotinylated antibody for 1 h. Followed by washing 5x, wells were incubated in 100 µl horseradish peroxidase-labeled streptavidin solution for 20 min in the dark at room temperature. Followed by another washing step 5x, 100 µl of chromogenic reagent TMB solution was added and allowed to react for 20 min at room temperature in the dark. Finally, 50 µl of stop solution was added and mixed well, followed by absorbance measurement at 450 nm under a microplate reader. The target protein concentration was calculated by plotting the standard curve graph.

### Hydrogen peroxide content assay

Oxidative stress was evaluated by determining the level of hydrogen peroxide (H_2_O_2_) in the serum samples using the hydrogen peroxide content assay kit (Solarbio, Beijing, China, Cat#BC3595). All the steps were performed according to the manufacturer’s protocol and finally, the samples were measured at the absorbance of 415 nm under BioTek Synergy 2 microplate reader.

### Total antioxidant capacity (T-AOC) assay

The antioxidant capacity was determined in serum samples using a total antioxidant capacity test kit (T-AOC) purchased from Solarbio, Beijing, China (Cat#BC1315) according to the manufacturer’s protocol. Four graded concentrations of ZLHXTY water extract, i.e., 0.07, 0.14, 0.28, and 0.42g per 1 ml of extraction solution, were also evaluated for their intrinsic antioxidant potential and all the samples were measured under BioTek Synergy 2 microplate reader at the absorbance of 593 nm.

### Nrf2 transcriptional factor activity assay

The transcriptional activity of Nrf2 was measured with RayBio^®^ Mouse Nrf2 TF-Activity Assay Kit (Catalog #: TFEM-Nrf2, Norcross, GA 30092, United States). Nuclear protein was extracted from the brain samples using a nuclear and cytoplasmic protein extraction kit according to the manufacturer’s instructions (Sangon Biotech, Shanghai, China, Cat# C510001). The procedures were performed following the manufacturer’s protocol.

### Quantitative real time polymerase chain reaction (qRT-PCR)

The qRT-PCR analysis for mRNA expression was performed for the following set of genes: Nfe2l2, Hmox1, Nqo1, Mgst1, and Sod1, the primer sequences of which are given in Table 3. Briefly, the brain tissues from the hemorrhagic cortex were homogenized in Trizol reagent (Beyotime Biotechnology, China, Cat# R0016) to extract total RNA. The absorbance was measured under a UV spectrophotometer at 260 and 280 nm, considering a ratio value > 1.8 for OD260/OD280 as appropriate for RNA sample quality. Reverse transcription of total RNA into cDNA was performed with HiScript III RT SuperMix for qPCR (+gDNA wiper) kit (Vazyme Biotech Co., Ltd, China, Cat. No. R323-01). The qRT-PCR was carried out in the presence of a fluorescent dye ChamQ universal SYBR qPCR Master Mix (Vazyme Biotech Co., Ltd, China, Cat. No. Q711-02/03) on the LightCycler^®^ 480 Instrument II (Roche, United States). The gene expression values were obtained through a cDNA standard curve. The mRNA levels were normalized to the GAPDH expression and calculated by the 2^−ΔΔCt^ method.

**TABLE 3:** Primer sequences (5′- 3′).

Gene name	Primer sequence	Product length
Nfe2l2	F: TCT​TGG​AGT​AAG​TCG​AGA​AGT​GT	23
	R: GTT​GAA​ACT​GAG​CGA​AAA​AGG​C	22
Sod1	F: GCC​CGC​TAA​GTG​CTG​AGT​C	19
	R: CCA​GAA​GGA​TAA​CGG​ATG​CCA	21
Nqo1	F: AGG​ATG​GGA​GGT​ACT​CGA​ATC	21
	R: AGG​CGT​CCT​TCC​TTA​TAT​GCT​A	22
Hmox1	F: AAG​CCG​AGA​ATG​CTG​AGT​TCA	21
	R: GCC​GTG​TAG​ATA​TGG​TAC​AAG​GA	23
Mgst1	F: CTC​AGG​CAG​CTC​ATG​GAC​AAT	21
	R: GTT​ATC​CTC​TGG​AAT​GCG​GTC	21
GAPDH	F: CGG​AGT​CAA​CGG​ATT​TGG​TCG​TAT	24
	R: AGC​CTT​CTC​CAT​GGT​GGT​GAA​GAC	24

### Western blot analysis

Samples of brain tissues were lysed and homogenized in RIPA buffer containing 1 mM PMSF on ice. The homogenized samples were centrifuged, and supernatants were collected in new eppendorf tubes. The supernatants containing protein were measured using a BCA protein concentration assay kit (enhanced) assay method (Beyotime, Shanghai, China, Cat#P0010S). Each sample containing 40 µg protein was prepared with loading buffer and subjected to electrophoresis on 12% SDS-PAGE gel, and then transferred to nitrocellulose membranes under appropriate voltage and time for specific proteins. The blots underwent blocking with 5% skimmed milk/TBST with gentle shaking for 1 h at room temperature. Later, the blots were incubated overnight with respective primary antibodies (listed in Table 4) with gentle shaking. The following day, after recovering the primary antibody solutions, the membranes were washed with TBST 5min x3, and subsequently incubated in Alexa Fluor 790 and Alexa Fluor 680 fluorescently labeled secondary antibodies (Invitrogen Life Technologies, United States, Cat#A11357 and A21109); at dilution ratio 1:10,000, for 1 h at room temperature. Membranes were then washed with TBST 5min x3 and observed under a laser scanner (Amersham Typhoon™, Cytiva, United States) for NIR fluorescent signal detection. The optical density was measured using the ImageJ software (NIH, Bethesda, MA, United States), and the values were normalized with respect to GAPDH.

**TABLE 4:** Primary antibodies.

Antibody	Type	Dilution	Uniprot RRIDs	Catalogue number	Source
NRF2(D1Z9C)XP	Monoclonal	1:1000	Q16236	12721	Cell Signaling Technologies Inc., Shanghai, China.
SQSTM1/p62 (D6M5X)	Monoclonal	1:1000	Q64337	23214	
Phospho-SQSTM1/p62(Ser349) (E7M1A)	Monoclonal	1:1000	Q13501	16177	
Keap1(F-10)	Monoclonal	1:1000	Q9Z2X8	sc-514914	Santa Cruz Biotechnology, Inc., CA, United States.
HO1(F-4)	Monoclonal	1:1000	P14901	sc-390991	
NQO1 (A180)	Monoclonal	1:1000	Q64669	sc-32793	
GAPDH	Monoclonal	1:10 000	P04406	AB0037	Abways Technology, Inc., Shanghai, China.

### Fluoro-jade C staining

Frozen brain tissues were cryo-sectioned and fixed in 4% paraformaldehyde for 30 min, with subsequent 5 min rehydration in 80%ethanol and NaOH solution mixed in a 9:1 ratio; followed by immersion in 70% ethanol and then distilled water, for 2 min in each. The blocking was carried out in KMnO_4_ solution diluted in distilled water (1:9 ratio), for 10 min. After blocking, the slides were rinsed with distilled water for 2 min, followed by fluoro-jade C staining (along with DAPI) for 10 min in the dark (Fluoro-Jade C RTD Stain Reagent, Biosensis, Australia, Cat#TR-100-FJ). Later, the slides were rinsed with distilled water for 1 min x 3. The slides were dried in an oven preheated at 50°C, for 5 min in the dark, and then dehydrated in xylene for 1 min. A drop of non-fluorescent mounting medium was placed on the tissue and clean coverslips were carefully placed over it. The slides were kept in the dark and observed under a Leica DM4 B fluorescence microscope equipped with a Leica DMC6200 camera. The photomicrographs were acquired at magnification ×200 (Objective lens ×20 and eyepiece lens 10x), using Leica application suite X software. The photomicrographs were adjusted for background and contrast using adobe photoshop software version 7.0.

### Statistical analysis

Results are expressed as mean ± S.D. Statistical analysis was performed by GraphPad Prism-5 statistic software Version 8.0.1 software (GraphPad Software Inc., La Jolla, CA, United States). The statistical comparisons were analyzed by ANOVA followed by Tukey *post hoc* test. Differences with *p < 0.05, **p < 0.005, and ***p < 0.001 were considered statistically significant.

## Results

### ZLHXTY capsule ameliorates the neurological outcome after ICH

**FIGURE 1:**
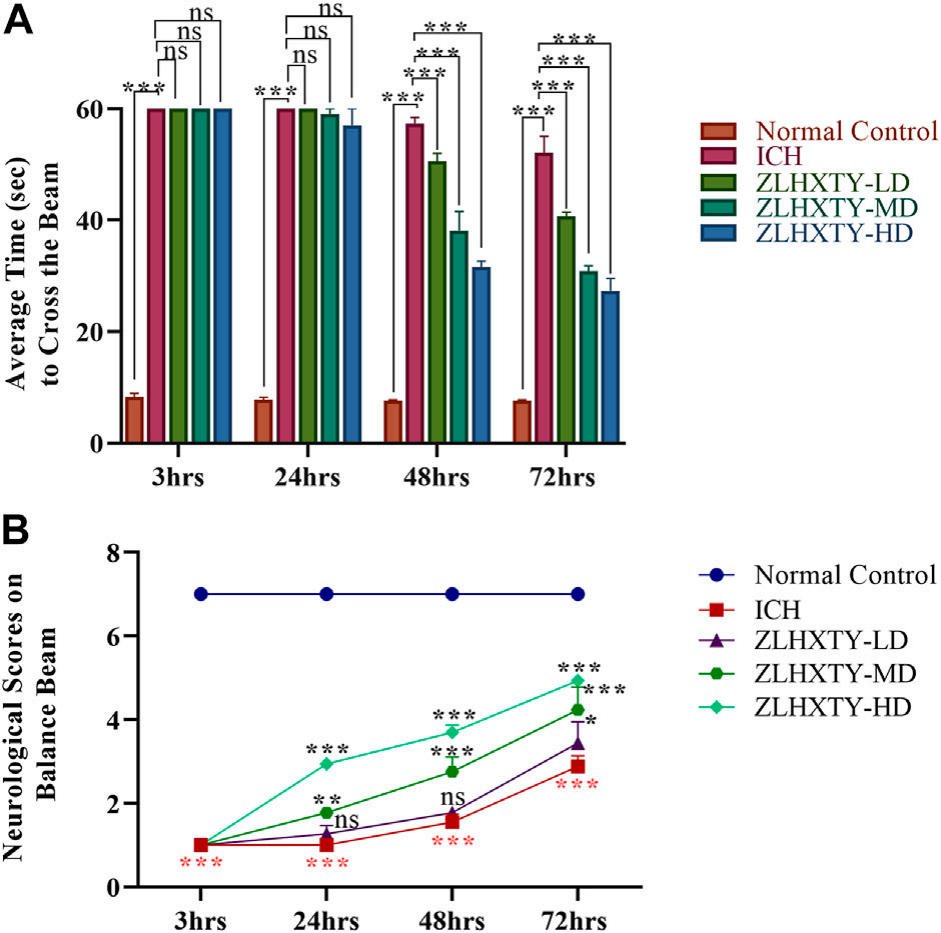
Neurological Scoring. **(A)** Balance beam test recorded as an average time to cross the beam within 60 s, showing significant improvement in LD, MD and HD ZLHXTY groups after 48 and 72 h. **(B)** Neurological scoring on the beam according to Feeny et al., indicating significant improvement in neurological score with MD and HD ZLHXTY at all the observed time points. Data represent the mean ± SD, n = 18, *p < 0.05, **p < 0.005, ***p < 0.001.

The balance beam test with neurological scoring was performed to evaluate the protective effect of ZLHXTY on the restoration of neurobehavioural outcomes after ICH. The ICH model animals demonstrated a complete inability to walk with significant fearfulness and shivering while on the beam when observed after 3 and 24 h, however, after 48 and 72 h animals showed to stumble on the beam with frequent paw slips (**p < 0.005, ***p < 0.001). The mice in 24 h ZLHXTY treatment group also demonstrated enhanced fear and frequent paw slips while limping on the beam, however, the overall behavior and the walking ability were significantly improved (***p < 0.001) after 48 h s and 72 h (Figure 1A). The behavioral score on the balance beam is shown in Figure 1B.

### ZLHXTY capsule reduces the inflammation after ICH

**FIGURE 2:**
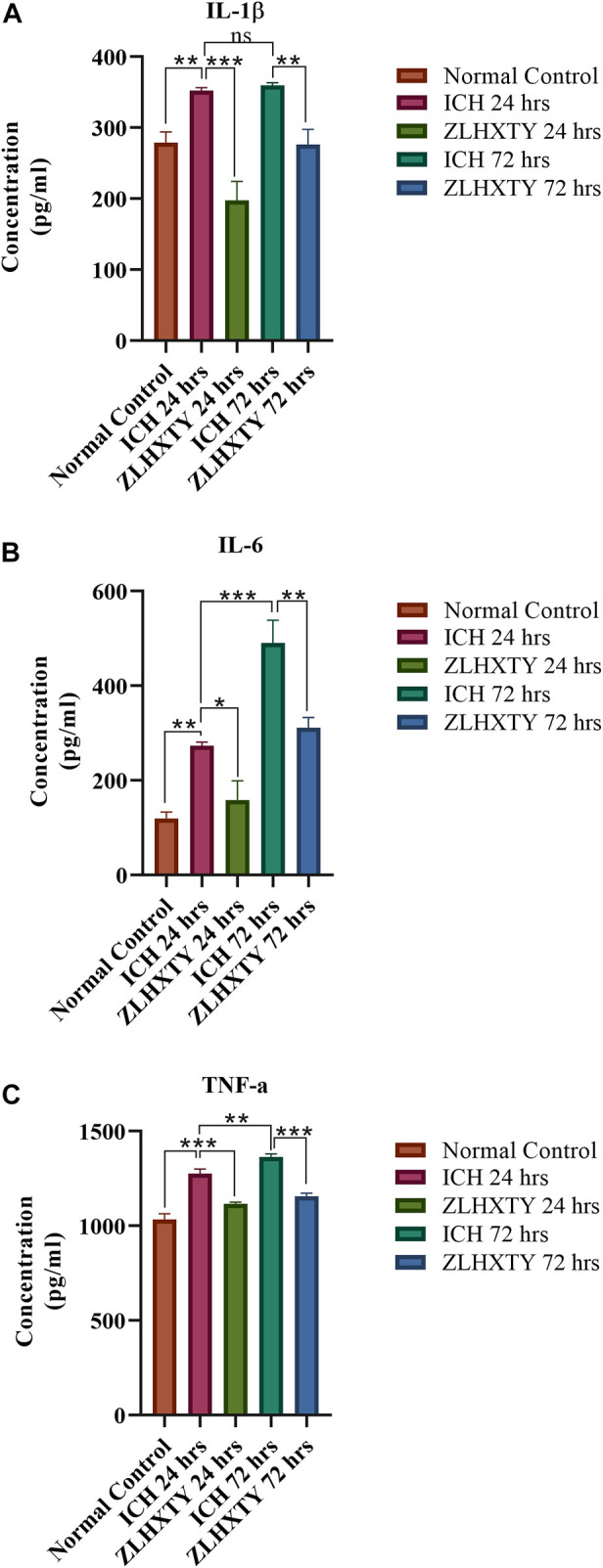
ELISA based cytokine detection. The presence of inflammatory cytokines in serum **(A)** IL-1β **(B)** IL-6 **(C)** TNF-α. Graphs show significantly raised values after 24 h of ICH with further increase after 72 h. ZLHXTY capsule treatment significantly lowered the elevated cytokines levels. Data represent the mean ± SD, n = 3, *p < 0.05, **p < 0.005, ***p < 0.001.

As identified previously, the treatment with ZLHXTY capsule after ICH protected from aggravated inflammatory brain injury by inhibition of the NFкB signaling pathway and its downstream targets (Mazhar et al., 2022). Similarly, the ELISA-based evaluation revealed significant elevation of inflammatory mediators IL-1β, IL-6, and TNF-α in the sera of ICH mice (**p < 0.005, ***p < 0.001) both after 24 and 72 h, as compared to normal mice. However, ZLHXTY treatment after 24 h and 72 h significantly lowered the serum levels of IL-1β, IL-6, and TNF-α as compared to the elevated expression observed after ICH (***p < 0.001) (Figures 2A–C).

### ZLHXTY capsule resist the oxidative stress after ICH

**FIGURE 3:**
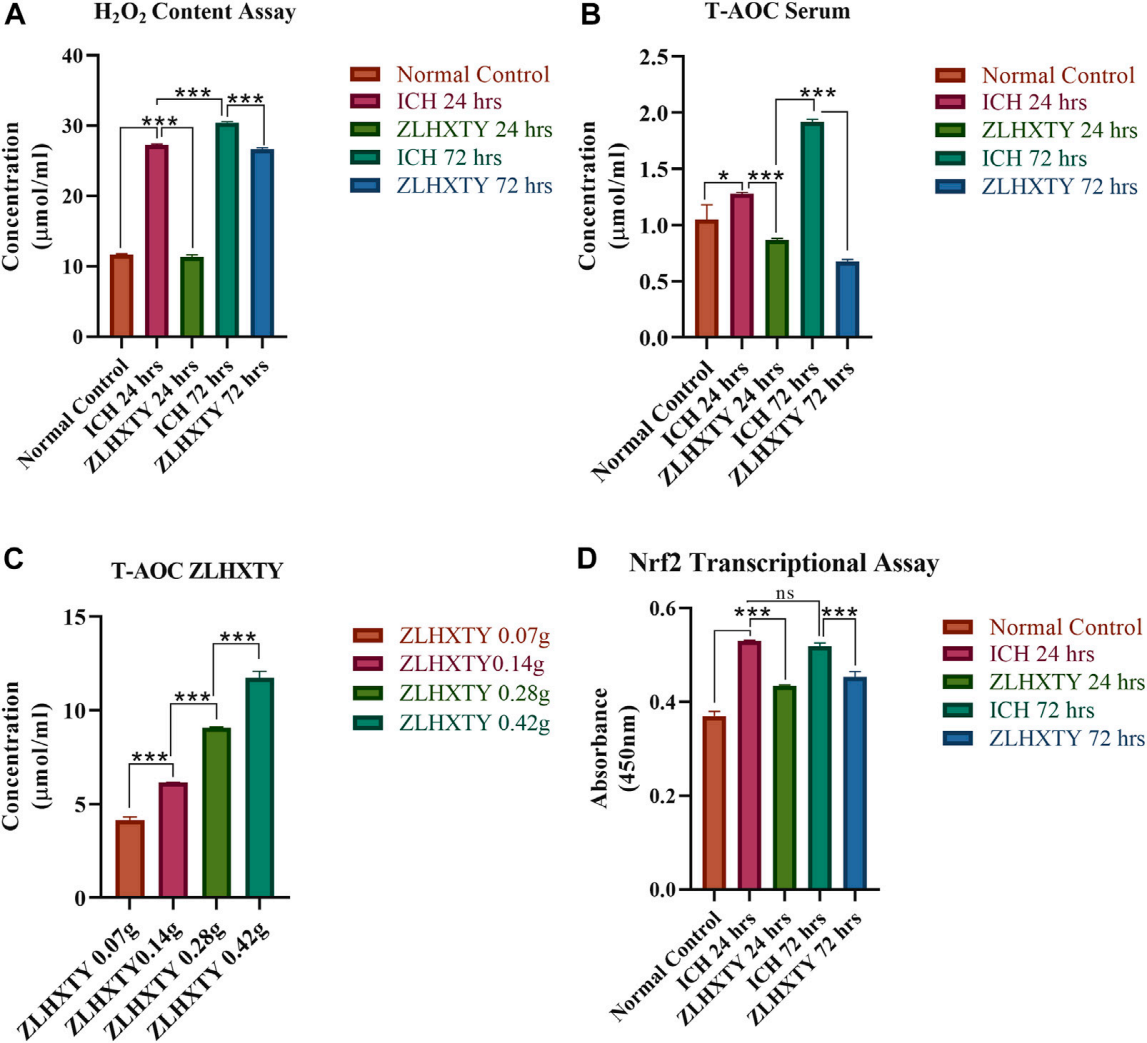
Oxidative and Antioxidant Assays. **(A)** H_2_O_2_ content assay of serum revealed significant elevation of hydrogenperoxide after 24 h with further increase after 72 h of ICH, which is reduced after ZLHXTY treatment. **(B)** Total antioxidant capacity assay revealed increased antioxidant capacity after ICH 24 h and further elevation at 72 h, while ZLHXTY treatment reduced the antioxidant capacity in serum. **(C)** Total antioxidant capacity of graded concentrations of ZLHXTY revealed increase in antioxidant capacity in a concentration dependent manner. **(D)** Nrf2 transcriptional assay revealed actively increased Nrf2 transcription after ICH which is downregulated to normal by ZLHXTY capsule. Data represent the mean ± SD, n = 3, *p < 0.05, **p < 0.005, ***p < 0.001.

During primary ICH injury, excessive generation of reactive oxygen species including hydrogen peroxide (H_2_O_2_) leads to oxidative stress, which is the key phenomenon leading to secondary damage resulting from the apoptosis, autophagy, inflammatory response, and blood-brain barrier (BBB) disruption (Yao et al., 2021). In our study, we found that the serum H_2_O_2_ content was significantly elevated after ICH (***p < 0.001) as compared to the normal group. Whereas, the ZLHXTY capsule significantly reduced the serum H_2_O_2_ content in the treatment groups (***p < 0.001). The results of an assay for hydrogen peroxide (H_2_O_2_) content as a marker of oxidative stress are shown in Figure 3A.

### ZLHXTY capsules possess intrinsic antioxidant potential

Supporting the results of H_2_O_2_ content that were significantly elevated after ICH, the results of the T-AOC assay revealed that antioxidant capacity was also increased after ICH (*p < 0.05 compared to normal). Inconsistently, after ZLHXTY treatment the serum antioxidant capacity was reduced as compared to ICH and the normal group (***p < 0.001) (Figure 3B). On the other hand, the four different concentrations of ZLHXTY water extract showed that ZLHXTY antioxidant potential increases with increasing concentration, indicating the intrinsic antioxidant potential of the ZLHXTY capsule (Figure 3C).

### ZLHXTY capsule regulates the Nrf2 transcriptional activity after ICH

It was observed that Nrf2 transcriptional factor activity was significantly upregulated after ICH induction after 24 h (***p < 0.001) as compared to the normal group, which was further exaggerated after 72 h non-significantly, in response to resulting oxidative and inflammatory stress. However, following ZLHXTY capsule treatment both at 24 h and 72 h, the Nrf2 transcription was restored to normal levels that might correlate with the intrinsic anti-oxidant potential of ZLHXTY capsules (***p < 0.001). This result further supports that the ZLHXTY capsule exerts its neuroprotection via its antioxidant property while modulating the Nrf2 antioxidant defense mechanism after ICH-induced oxidative stress (Figure 3D).

### ZLHXTY capsule normalizes the mRNA levels of Nrf2 and its downstream antioxidant target genes

**FIGURE 4:**
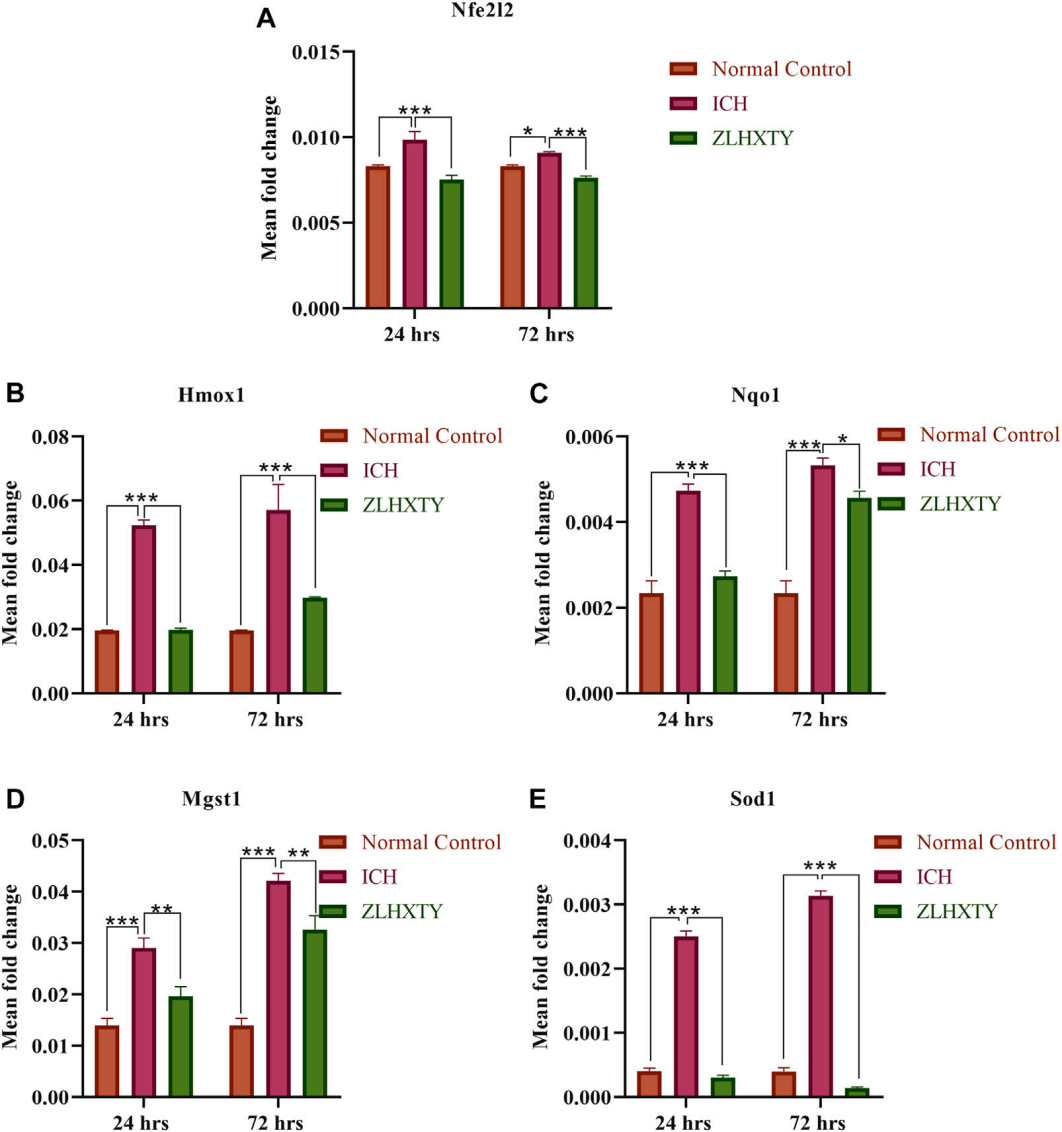
RT-qPCR **(A)**Nfe2l2 **(B)** Hmox1 **(C)** Nqo1 **(D)** Mgst1 **(E)** Sod1, showing significant elevation after 24 h with further increase after 72 h of ICH whereas, ZLHXTY capsule treatment caused downregulation. Data represent the mean ± SD, n = 9, *p < 0.05, **p < 0.005, ***p < 0.001.

The mRNA expression levels of Nfe2l2 coding for transcription factor Nrf2, and its downstream antioxidant target genes such as Nqo1, Hmox1, Sod1, and Mgst1 were tested. After 24 h of ICH, the measured values of Nfe2l2, Nqo1, Hmox1, Sod1, and Mgst1 were significantly elevated as compared to the normal control group (***p < 0.001), which continued to be elevated until after 72 h of ICH. However, following ZLHXTY treatment both after 24 and 72 h have been shown to reduce their expression down towards normal (***p < 0.001 as compared to the ICH group), (Figures 4A–E).

### ZLHXTY capsules regulation of Nrf2 signaling revealed by protein expression

**FIGURE 5:**
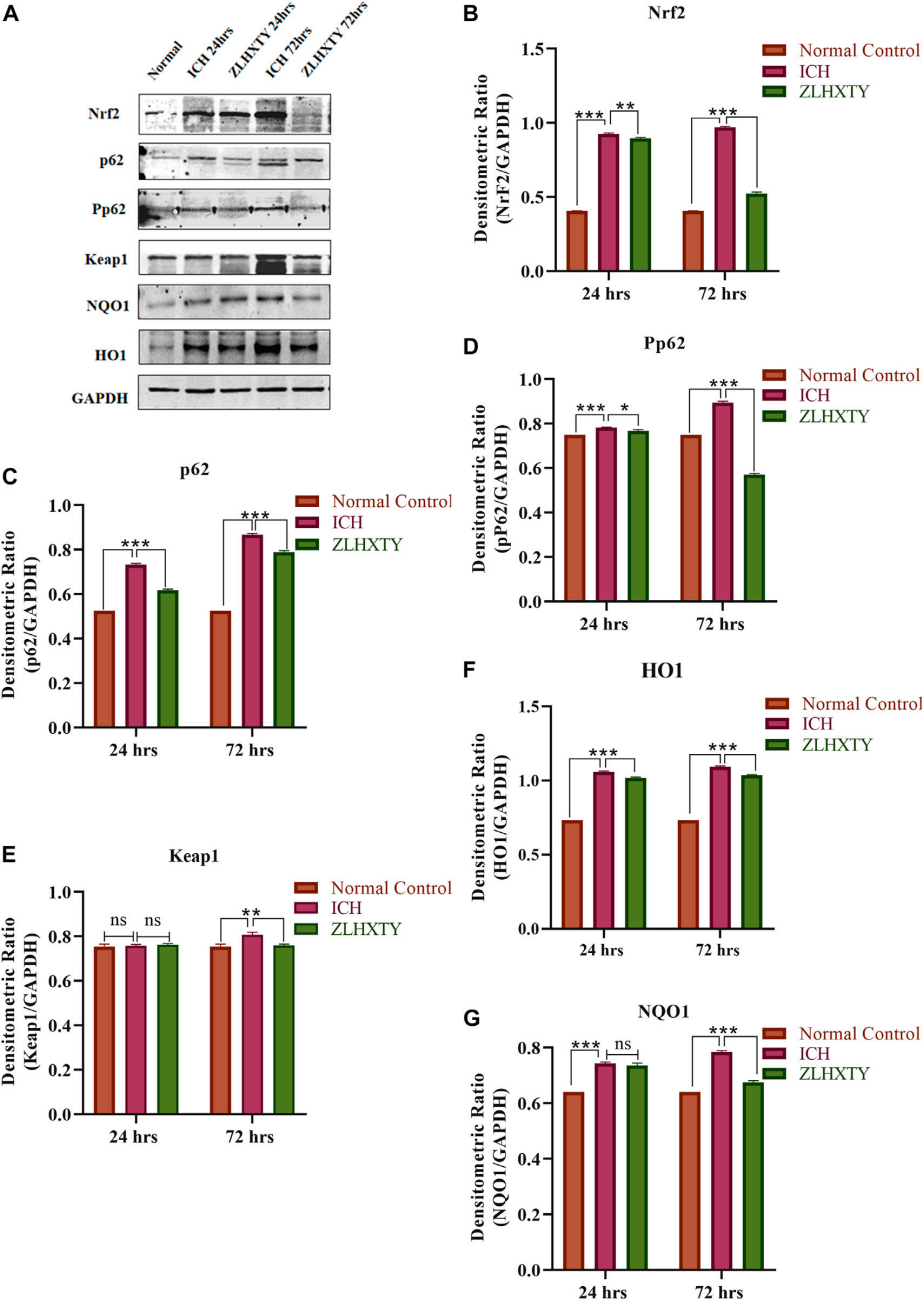
Western blotting **(A)** Representative immunoblots; graphical representation of measured densitometric ratio of specific proteins and GAPDH **(B)** Nrf2 **(C)** p62 **(D)** Pp62 **(E)** Keap1 **(F)** HO1 **(G)** NQO1, showing significant increase in expression after 24 h and farther after 72 h of ICH followed by regulatory effect of ZLHXTY capsule treatment. Data represent the mean ± SD, n = 3, *p < 0.05, **p < 0.005, ***p < 0.001.

The Western blot analysis was performed for analysis of protein expression of Nrf2, p62, Pp62, Keap, and downstream antioxidant proteins HO1 and NQO1, shown in Figures 5A–G. After 24 h of ICH, the protein expression of Nrf2 and p62 were significantly elevated (***p < 0.001) as compared to the normal group. However, after ZLHXTY treatment for 24 h, the Nrf2 expression was significantly decreased (**p < 0.005), as well as the expression of p62 was also lowered more significantly (***p < 0.001). The expression of p62 is supported by the parallel expression of phosphorylated p62, Pp62, indicating an active form of p62 regulating the ICH and ZLHXTY action mechanism. In contrast, the levels of keap1 were maintained at the same level in all the groups without significant alteration except in ICH 72 h group where it was slightly elevated. The expression pattern of downstream targets NQO1 and HO1 follow similarity with Nrf2 expression, i.e., significant elevation (***p < 0.001) after 24 h of ICH and downregulation after ZLHXTY 24 h treatment. However, all the proteins such as Nrf2, p62, Pp62, HO1, and NQO1 show significantly higher expression (***p < 0.001) after 72 h of ICH indicating the critical time point for regulation of disease mechanism. The effect was regulated by ZLHXTY treatment for 72 h, decreasing the protein expression to resume normal levels.

### ZLHXTY capsules control neuronal damage

**FIGURE 6:**
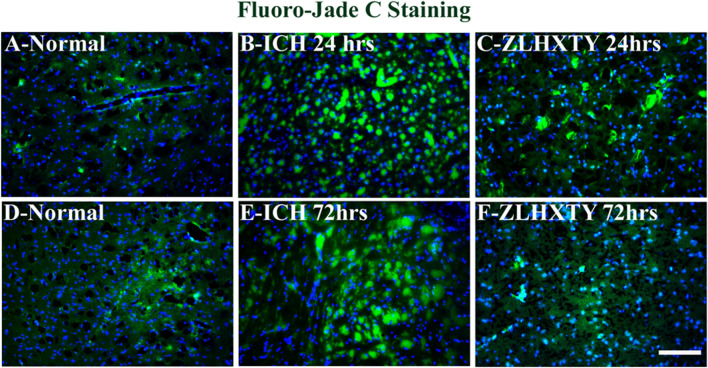
Fluoro-Jade C Staining. Brain sections stained with Fluoro-Jade C (green) to identify neuronal damage, and DAPI (blue) to mark nucleus. **(A, D)** Normal brain sections, without any sign of neuronal damage. **(B)** ICH 24 h group reveal obvious neuroal damage with increased number and signal of green fluorescence. **(C)** ZLHXTY 24 h group show significant control over neuronal damage. **(E)** ICH 72 h show excessive neuronal damage, even higher than that observed in ICH 24 h group. **(F)** ZLHXTY 72 h group show only few damaged neuronal cells, indicating significant control over neuronal damage. 50µm scale bar corresponds to ×200 magnification.

The neuronal damage was evaluated by using Fluoro-jade C staining on brain tissue sections. In normal tissues (Figures 6A,D) there was no sign of neuronal damage in the brain tissue slides around the basal ganglia region in the right hemisphere. After 24 h of ICH, the neuronal damage in the perihematomal region was obvious, and an increase in the green fluorescent signals was observed (Figure 6B). However, after ZLHXTY treatment for 24 h, the neuronal damage was significantly controlled as compared to ICH 24 h model (Figure 6C). The extent of neuronal damage was markedly increased even higher than that after 24 h of ICH injury as evidenced by the increased number of green fluorescent signals in the brain tissue (Figure 6E). Following ZLHXTY treatment for 72 h, the neuronal damage was significantly controlled and few to negligible green fluorescent signals were found as an indicator of neuronal damage (Figure 6F).

## Discussion

Oxidative stress following intracerebral hemorrhage is a key phenomenon underlying aggravated brain injury. The redox imbalance due to the excessive production of reactive oxygen species activates antioxidant defense pathways such as Nrf2 signaling to combat the oxidative stress (Yao et al., 2021). In the present study, we tested the hypothesis that ZLHXTY capsules alleviate intracerebral hemorrhage-induced brain injury in a mouse model by combating oxidative stress and inflammation, on its early administration, i.e., within 2 h of ICH induction. It is presumed that the neuroprotective effect of ZLHXTY capsules may partially involve its intrinsic antioxidant potential that regulates redox imbalance and maintain Nrf2 transcription and antioxidant response elements after ICH. This study is further proof of evidence to our previous research on exploring the anti-inflammatory role of ZLHXTY capsules in inhibiting TNFα-NFκB canonical signaling after ICH.

Oxidative stress damage mediated by reactive oxygen species-mediated plays a crucial role in deciding the course of disease in severe brain injury in humans (Paolin et al., 2002). A similar finding was also evidenced in our experiment indicating poor neurological outcomes after ICH due to a high oxidative stress environment. Accumulated research evidence indicates a direct relationship between high ROS/oxidative stress in plasma/serum and poor neurological outcome/mortality after traumatic brain injury (Nayak et al., 2008; Hohl et al., 2012; Lorente et al., 2019; Park et al., 2022). Maintaining antioxidant levels can effectively control the oxidative stress that induces damage and therefore, improve the neurological outcome (Park et al., 2022), as is also observed after ZLHXTY capsule treatment after ICH in our experiment.

Excessive production of reactive oxygen species is known to progress primary hemorrhage into aggravated secondary injury. In our experiments, we also observed significant ROS production through hydrogen peroxide content assay after 24 and 72 h of ICH, which was subsided by ZLHXTY capsule treatment. Soon after hemorrhage, the infiltration of reactive leukocytes and microglia/macrophages secreting various cytokines and chemokines, act as a major source of ROS causing damage to perihematoma tissue (Zhou et al., 2014). Available data from both clinical and preclinical resources support the contribution of activated leukocytes and microglia/macrophages in the progression of ICH-induced early brain injury (Keep et al., 2012). In addition, hemoglobin and its metabolites: iron, carbon monoxide, and biliverdin; released via erythrolysis further contribute to ICH brain injury (Keep et al., 2012). Subsequently, iron accumulation in the brain leads to abundant ROS generation via Fenton reaction, causing neurotoxicity (Xiong et al., 2014). Together these mechanisms interact resulting in disruption of the blood-brain barrier (BBB), neuronal damage, and gliosis with lasting neurological deficits (Hu et al., 2016).

To further confirm our result, we performed a total antioxidant capacity assay and observed that ZLHXTY capsules possess intrinsic antioxidant activity which increases with increasing concentration. This can be further explained by the constitution of the ZLHXTY capsule, which is a mixture of five Chinese medicines derived from natural origin with multiple constituents and rich antioxidant potential. Extensive research studies have identified the antioxidant properties of *A. membranaceus* Fisch. ex Bunge (Durazzo et al., 2021; Samuel et al., 2021), *C. cassia* L.) J. Presl (Yang et al., 2012; Rao and Gan, 2014), and *S. cuneata* (Oliv.) Rehder and E.H. Wilson (Zhang et al., 2021), displaying significant prevention from tissue injury via antioxidant mechanisms. In addition, two constituents of the ZLHXTY capsule are of animal origin, i.e., *H. nipponica* Whitman and *P. aspergillum* (E. Perrier), that have also been shown to act through antioxidant mechanisms (Li et al., 2021; Xu et al., 2022).

As explained in a previous study, the absorption of antioxidants after their consumption must be reflected as the increment in total antioxidant capacity in serum. However, the total antioxidant capacity in plasma/serum is under strict regulatory control and the increased levels usually reflect the adaptation to increased oxidative stress (Lorente et al., 2015; Morimoto et al., 2019). Similarly, we observed an increased T-AOC in the serum of ICH groups both at 24 and 72 h, probably as a physiological mechanism to counter the increased ROS production. However, the ZLHXTY treatment decreased the total antioxidant capacity more than the ICH groups. Elevated serum antioxidant capacity may not necessarily be a desirable condition, probably indicating an underlying ongoing pathological process in the body, as yet the decreased levels might be in response to decreased production of reactive species *in vivo* (Ogawa et al., 2011; Lorente et al., 2015; Morimoto et al., 2019). Therefore, as a net result of the two methods, i.e., hydrogen peroxide content and total antioxidant capacity, we can assume that ZLHXTY maintains the redox imbalance and counter the ROS production after ICH.

Nrf2 has a crucial function in limiting the cascade of events originating from oxidative stress, leading to ICH-induced early brain injury. Nrf2 is a major transcription factor responsible to induce phase II detoxification enzymes through upregulation of antioxidant response element (ARE) mediated antioxidant genes expression such as NAD(P)H: quinone oxidoreductase 1 (NQO1), heme oxygenase 1 (HO-1), glutathione s-transferase (GST), glutamylcysteine ligase (the rate-limiting enzyme in glutathione synthesis), thioredoxin reductase 1, and thioredoxin (Keep et al., 2012). Accumulated research reported that Nrf2 expression gradually increased within 2 h following ICH, reaching its peak at 24 h, mediating the induction of antioxidant and detoxification enzymes/proteins expression, resulting in an improved neurological outcome, alleviated cerebral edema, and decreased inflammation (Shang et al., 2013). We also identified the same observation of increased Nrf2 transcriptional activity after 24 h and further increment at 72 h of ICH, which was further confirmed through RT-qPCR and Western blot. The downstream antioxidant targets, i.e., HO1, NQO1 nad MGST1 were also expressed concordantly as Nrf2, as observed at the transcriptional and protein levels. Previous studies have also documented the protective role of the Nrf2 gene, by identifying more neuronal vulnerability to oxidative stress and aggravated brain damage following ICH in Nrf2^−/−^ mice, compared with WT mice, because of decreased NQO1 and GST activities (Kensler et al., 2006). Furthermore, neuroblastoma cell lines with Nrf2 silencing or gene knockout were found to be more susceptible to apoptosis than the vector-only transfected cells, due to the suppression of ARE-mediated genes (Zhao et al., 2006).

Nrf2 activation is regulated by two mechanisms, i.e., canonical and non-canonical mechanisms. During basal conditions, Nrf2 levels are mainly regulated by Kelch-like ECH-associated protein 1 (Keap1) i.e., E3 ubiquitin ligase substrate adaptor, mediating Nrf2 polyubiquitination and constant degradation through the proteasomal pathway. However, some molecules of Nrf2 evade Keap1-dependent degradation and translocate into the nucleus generating a constitutive and weak signal of target genes expression. This ubiquitin ligase activity of Keap1-dependent Nrf2 degradation is regulated in a redox-sensitive manner. During oxidative stress or in the presence of electrophilic compounds, a conformational change occurs at specific cysteine residues of Keap1 that are oxidized, causing inhibition of the E3 ubiquitin ligase activity. This inhibition of Keap activity allows newly synthesized Nrf2 molecules to translocate into the nucleus and bind to the ARE for induction of target gene expression. This mechanism is called the canonical activation of Nrf2, which requires the oxidation of cysteine residues of Keap1 (Hennig et al., 2018). However, Nrf2 activation upon the disruption of Keap1-Nrf2 complex by phosphoactivation of p62 and some other proteins such as DPP3, WTX, PALB2, p21, and BRCA1 is termed as non-canonical Nrf2 activation (Silva-Islas and Maldonado, 2018). In our experiment, it is observed that both the transcriptional and protein expression of p62 and Pp62 was found to be upregulated after ICH but not the Keap transcriptional and protein expression in response to increased Nrf2 activity. A ubiquitin-binding protein p62, serves as a cargo receptor during autophagy (Katsuragi et al., 2016). Under normal physiological conditions, p62 interacts with autophagic ubiquitinated substrates and delivers them to autophagosomes for degradation. However, under conditions of oxidative stress when the autophagy is impaired, p62 gets accumulated in the cytoplasm and gets phosphorylated for selective binding to the ubiquitinated autophagic cargos (such as damaged mitochondria, protein aggregates, and invasive bacteria). When p62 is phosphorylated at S351 of the KIR, in an mTORC1-dependent manner, its affinity for Keap1 increases, leading to sequestration of Keap1 on autophagic cargos for degradation. Consequently, Nrf2 molecules are stabilized allowing more molecules to translocate into the nucleus and bind to the target site on DNA for induction of cytoprotective target genes (Lau et al., 2010; Ichimura et al., 2013; Ahmed et al., 2017). Since p62 is a target of Nrf2, there exists a positive feedback loop in the p62-Keap1-Nrf2 axis. Moreover, p62 also regulates NF-κB expression that, successively, increases Nrf2 expression (Hennig et al., 2018).

Our results demonstrated the downregulation of Nrf2 and its downstream antioxidant targets to normal levels in ICH animals that have undergone treatment with ZLHXTY capsule possessing intrinsic antioxidant activity. To explain this, we assume that an autoregulatory feedback loop mechanism in the Nrf2 pathway was activated upon antioxidant exposure where excessive Nrf2 leads to an increase in INrf2 (Keap1) gene expression that leads to ubiquitination and degradation of Nrf2 reducing its levels to normal (Lee et al., 2007).

## Conclusion

To conclude, we have demonstrated that ZLHXTY capsules hold antioxidant potential through which it can improve neurological outcomes, and inhibit redox imbalance and inflammation following ICH injury as shown by its modulation of Nrf2 signaling.

## Data Availability

The raw data supporting the conclusion of this article will be made available by the authors, without undue reservation.

## Supplementary material

SUPPLEMENTARY FIGURE S1 UPLC-HRMS chromatogram for ZLHXTY capsule characterization. **(A)** ZLHXTY capsule chromatogram in positive ion modes, m/z range 150-1500, resolution 70,000; **(B)** Chemical standards; L-epicatechin, calycosin-7-O-β-glucoside, coumarin, ononin, calycosin, cinnamaldehyde, formononetin, and wogonin.
