# Supplementary figures and images for: Zhilong Huoxue Tongyu capsule attenuates intracerebral hemorrhage induced redox imbalance by modulation of Nrf2 signaling pathway
Source: Front Pharmacol. 2023 Jun 7;14:1197433. doi: 10.3389/fphar.2023.1197433 (PMC10282143; doi:10.3389/fphar.2023.1197433)

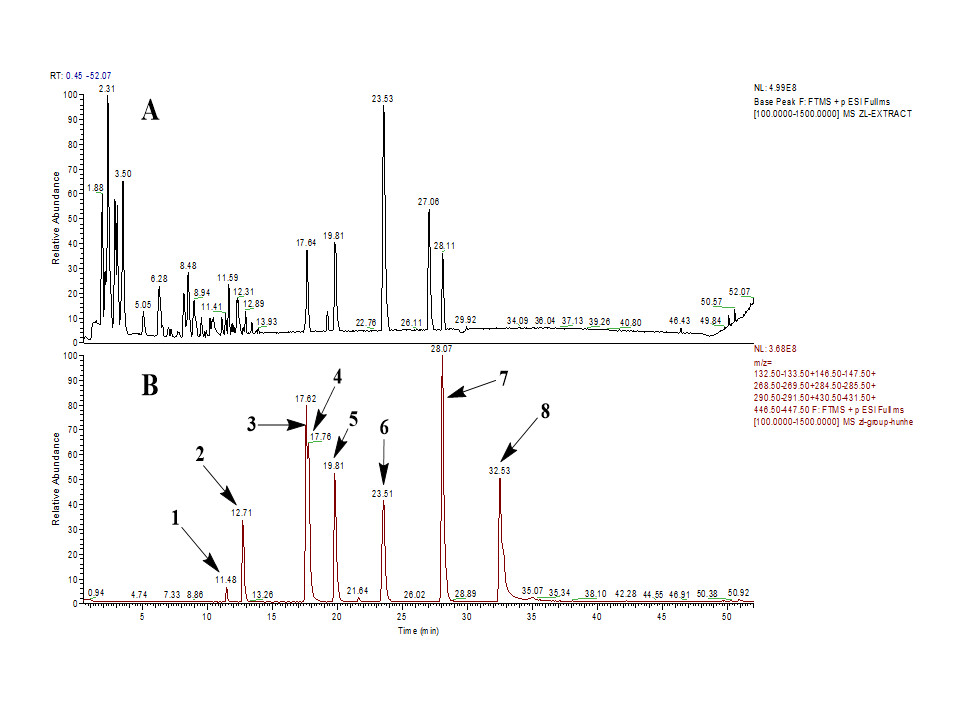

Supplement: Supplementary file 2 [file Image1.tif]
